# Artificial intelligence in cancer-related malnutrition and cachexia: a transformative tool in clinical nutrition

**DOI:** 10.1016/j.advnut.2025.100447

**Published:** 2025-05-13

**Authors:** Salvatore Carbone

**Affiliations:** 1 Nutrition Program, EVMS School of Health Professions, Macon & Joan Brock Virginia Health Sciences at Old Dominion University, Norfolk, VA, USA; 2 Division of Endocrine and Metabolic Disorders, Strelitz Diabetes Center, Department of Medicine, Eastern Virginia Medical School, Macon & Joan Brock Virginia Health Sciences at Old Dominion University, Norfolk, VA, USA

## Introduction

Malnutrition and cachexia are common complications in cancer patients, and they negatively influence prognosis, treatment efficacy, and tolerability as well as quality of life [[1], [2], [3]]. Accurately identifying and effectively managing malnutrition and cachexia in this population remains a clinical challenge. Conventional validated screening tools may lack the sensitivity and specificity required for early detection and personalized intervention in diverse cancer types and treatment settings [4,5]. Over the last decade, the use of artificial intelligence (AI), including machine learning (ML) and deep learning (DL) strategies, has shown promising results in clinical nutrition, with the potential to revolutionize nutritional care by providing more precise and scalable tools [6].

In the recent issue of the *Journal*, Sguanci et al. [7] investigated the role of AI in identifying and managing malnutrition and cachexia in cancer patients. The authors conducted a systematic review involving over 52,000 individuals to investigate AI’s potential for nutritional assessment, body composition monitoring, dietary adherence, and clinical outcomes in patients with cancer.

## AI-Enhanced Early Identification of Malnutrition

Timely and accurate identification of malnutrition risk is the cornerstone of effective nutrition therapy, including nutrition support. Sguanci et al. [7] highlighted the enhanced capabilities of AI models in predicting malnutrition compared with traditional screening methods. They demonstrated that ML algorithms had a superior predictive accuracy, consistently achieving AUC values exceeding 0.80, even without more typical weight loss markers. This early identification of malnutrition would allow clinicians to implement targeted nutritional interventions sooner in the disease trajectory, potentially preventing the development of severe complications typically associated with malnutrition. Moreover, AI was able to predict feeding tube dependence in patients with oral and oropharyngeal cancer. Furthermore, when bioelectrical impedance analysis-derived phase angle was included in the analysis, AI was able to further enhance malnutrition screening [7].

These results suggest that integrating AI into clinical workflows has the potential to simplify the early identification of malnutrition, but also to reduce the burden on clinical staff responsible for conducting malnutrition screening.

## The Identification of Cachexia and Treatment-responsive Cachexia With AI

Cancer cachexia, a clinical syndrome characterized by involuntary weight loss associated with both muscle mass and fat mass losses and functional decline, is a strong negative prognostic factor in patients with cancer, with extremely limited therapeutic targets. Only recently, a humanized monoclonal antibody inhibiting the growth differentiation factor (GDF)-15 has been shown to increase weight gain and physical activity as well as cachexia-related symptoms in this population [3]. Therefore, accurate assessment and monitoring of body composition are essential for identifying patients with cachexia early and evaluating the efficacy of both dietary and pharmacologic interventions.

Sguanci et al. [7] highlighted the role of DL models for accurate body composition analysis. These DL models have shown high segmentation accuracy (dice similarity coefficients: 0.92–0.94) for muscle and adipose tissue. This capability is particularly valuable for the early detection of sarcopenia and cachexia. Importantly, AI implementation was able to differentiate potentially reversible cachexia from refractory forms of cachexia with AUC values exceeding 0.80 [7]. This assessment could potentially allow for allocating resources more efficiently and perhaps identify those patients who might benefit the most from a nutritional intervention.

## Clinical Integration of AI and Impact on Patients’ Dietary Adherence and Satisfaction

The effects of the practical application of AI in oncology nutrition on clinical outcomes have been largely unexplored; however, we could speculate that by improving adherence to dietary interventions, patients will experience improvements in clinical outcomes.

Sguanci et al. [7] investigated the effects of AI-driven virtual dietitian systems designed to support patients with personalized nutritional therapy. Such platforms resulted in high rates of adherence to dietary recommendations (∼ 84%), which was associated with high patient satisfaction (∼94%), indicating their potential to improve the patient experience and overall adherence with nutritional care [7]. Additionally, AI-enhanced administrative tools demonstrated the capacity to reduce referral times for dietitian consultations by an average of 2.4 d, with an impressive predictive accuracy in identifying patients needing nutrition support (<10% misclassification rate) [7].

Although conclusive evidence on the impact of AI-driven virtual dietitian systems on hard clinical outcomes, such as survival and length of stay, is still being investigated, improvements in nutritional status, adherence to interventions, patient satisfaction, and timely support are clearly required steps to improve patient outcomes.

## Challenges and Future Directions

The integration of AI into routine oncology nutrition practice remains limited. This is likely caused by the significant heterogeneity across studies in terms of methodologies, patient populations, and the quality and standardization of datasets used to train AI models [7]. This level of heterogeneity makes it hard to replicate these findings across centers with different characteristics. Moreover, the use of AI in clinical practice requires critical ethical considerations, including data privacy and the potential for algorithmic bias, requiring utmost attention to build trust and ensure equitable application of AI technologies. Promoting transparency in AI models, often referred to as Explainable AI (XAI), is also a crucial aspect for clinicians to understand how AI-driven recommendations are generated and to maintain human oversight in clinical decision-making.

To fully leverage AI’s potential in clinical nutrition, future research must standardize methodologies and develop high-quality, diverse datasets for model training and validation. Rigorous prospective studies and randomized controlled trials are needed to conclusively evaluate the impact of AI-driven nutritional interventions on patient-centered outcomes, including survival, length of stay, quality of life, and functional status. Furthermore, exploring the cost-effectiveness and scalability of implementing AI solutions in a variety of healthcare environments with different resources is essential before widespread adoption.

**Figure 1:**
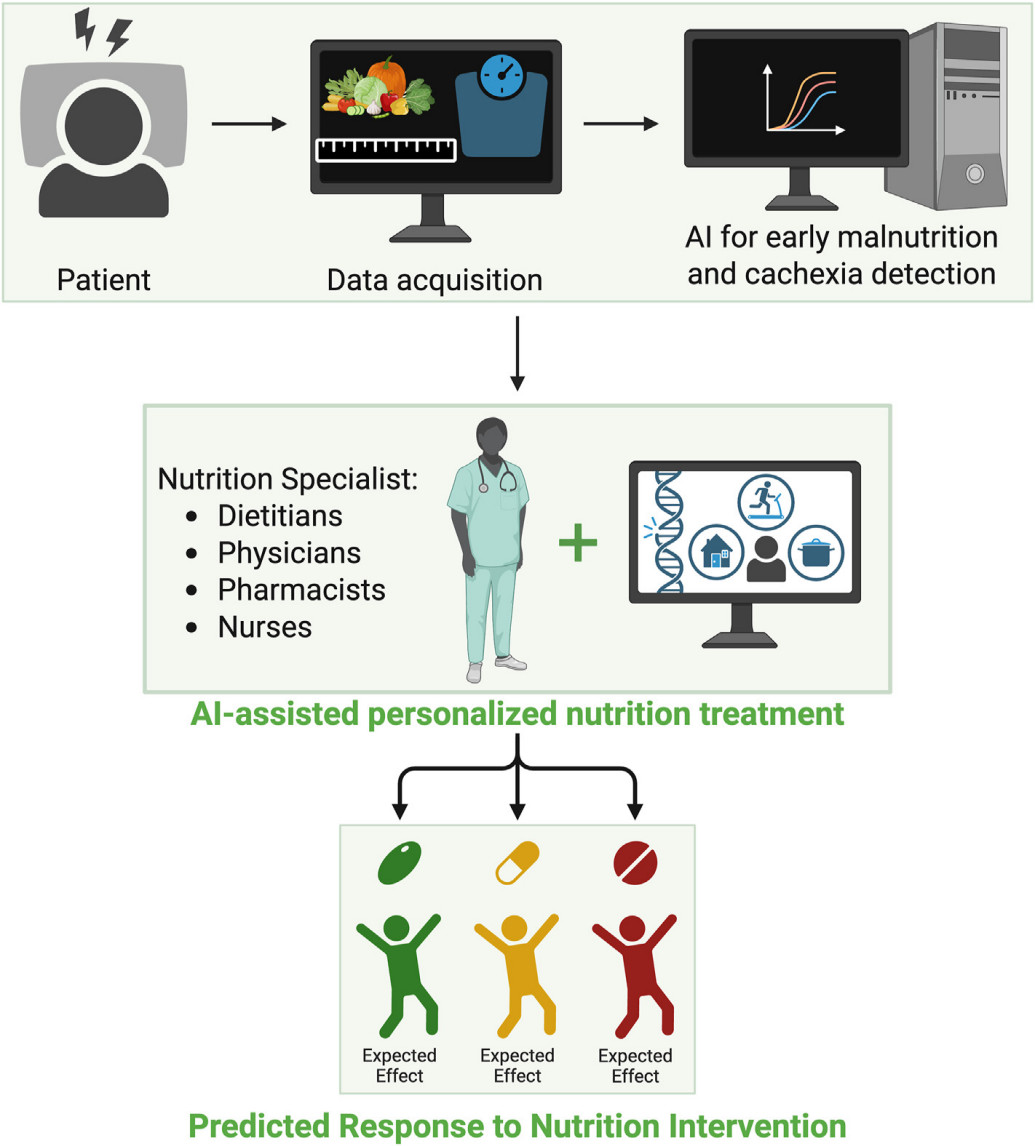
Potential role of artificial intelligence in early detection, treatment, and monitoring of malnutrition and cachexia. AI, artificial intelligence. Created in BioRender. Carbone, S. (2025) https://BioRender.com/v9vsr6q.

The future of oncology nutrition will require a synergistic partnership between clinicians and AI. AI tools can empower registered dietitian nutritionists and other nutrition specialists with enhanced analytical capabilities and predictive insights, allowing them to focus on patients who are more likely to respond to a nutrition intervention, ultimately providing a more personalized and effective care (Figure 1). The goal of AI is not to replace the clinician and their clinical judgment, but to support their ability to identify and treat malnutrition, cachexia, and other nutrition status abnormalities, to ultimately improve the lives of our patients.

## Author contributions

The sole author was responsible for all aspects of this manuscript.

## Declaration of generative AI and AI-assisted technologies in the writing process

During the preparation of this work, the author used Gemini (Google) to assist with improving the readability of the manuscript draft. After using this tool/service, the author reviewed and edited the content as needed and takes full responsibility for the content of the publication.

## Funding

The author reported no funding received for this study.

## Conflict of interest

The author reports no conflicts of interest.
